# Artificial intelligence in interdisciplinary life science and drug discovery research

**DOI:** 10.2144/fsoa-2022-0010

**Published:** 2022-03-08

**Authors:** Jürgen Bajorath

**Affiliations:** 1 Department of Life Science Informatics & Data Science, B-IT, LIMES Program Unit Chemical Biology & Medicinal Chemistry, Rheinische Friedrich-Wilhelms-Universität, Friedrich-Hirzebruch-Allee 5/6, Bonn, D-53115, Germany

**Keywords:** artificial intelligence, deep learning, drug discovery, experimental impact, interdisciplinary research, life sciences

Artificial intelligence (AI) is lauded as an auspicious problem solver in many areas. However, the methodology is not always sufficiently understood. In fact, there often is an aura of mystery – and also concern – created around AI. For example, the expectation that machines would ‘think’ independently and reach autonomous decisions beyond human reasoning, which might also be threatening, depending on the situation, is not factual and falls into the realm of science fiction. In science, the popularity and promise of AI mostly originate from notable advances in a few fields, but are also influenced by business-driven hype and partly unrealistic expectations.

In computer science, various disciplines are covered under the term AI [1]. Among these, deep learning (DL) using deep neural networks (DNN) – a sub-discipline of machine learning (ML) – has been responsible for recent progress in computer vision (image analysis) or natural language processing. These advances have greatly contributed to the popularity of AI in science. Robotics, another AI discipline, is a mainstay in industry and also plays an important role in laboratory automation. Furthermore, expert and recommender systems, which are also a part of AI, are beginning to be explored in the natural sciences. In the life sciences including medicinal chemistry and early-phase drug discovery, the focal points of this discussion, DL clearly dominates [2–5], and the term AI is for the most part synonymously used with DL. In medicine, a similar dominance of DL is observed across different therapeutic areas [6] such as radiology or oncology [7,8]. In clinical practice, medical image analysis represents a prime growth area for DL [7,9].

## Deep machine learning

Generally, ML uses algorithms for the extraction of feature patterns from training data to classify test objects or address regression tasks. Hence, ML methods are statistical in nature and derive predictive models capturing linear or non-linear instance–feature relationships based on inference from data. DNNs contain varying (and typically large) numbers of ‘hidden’ layers formed by mathematical ‘neurons’ and are particularly well suited for feature extraction from large volumes of unstructured data (such as pixels in images) and for learning new object representations, which represents a hallmark of DNNs. Using these computational architectures, DL relies on systematic correlation of feature patterns and known object labels (class labels) and derives models with decision functions that are not pre-programmed. ML/DL model performance is primarily assessed in benchmark studies using training and test data with known class labels. DL tends to outperform other ML methods in areas where large volumes of unstructured or low-resolution data are available. Hence, all in all, there is nothing mysterious about this type of supervised ‘machine intelligence’.

## Neural network characteristics

Shallow NNs were popular during the early stages of ML in biology, chemistry and drug discovery, but were largely replaced over time by other approaches such as decision tree methods (random forest and gradient boosting), Bayesian modeling or support vector machines. Reasons for this included a general tendency of shallow NNs to overfit models to training data and their high sensitivity to varying parameter settings. As second-generation NNs, DNNs then received increasing attention over the past decade. A part of the attraction is that DNNs represent highly versatile computational architectures. In computer science, a great variety of DNNs and associated learning strategies have been introduced, sometimes described with terms like a network ‘jungle’ or ‘zoo’ [10]. Hence, for many applications, alternative DNNs can be considered, but finding preferred solutions is not necessarily straightforward. Compared with other ML approaches, DNNs are particularly rich in hyper-parameters and derivation of DNN models requires substantial knowledge, skills and experience. Given the variety of DNN architectures and their many parameters, DNNs are currently not yet as extensively explored as other ML approaches. Accordingly, although public domain software is available for constructing DNNs, DL is not an approach that is readily accessible to non-experts. There is a strong discrepancy between the mechanics of model building, which might be handled by less experienced users, and evaluation of results and recognition of potential caveats or model errors, which requires much more expertise. Importantly, similar to other – but not all – ML methods, DNNs have notorious ‘black box’ character [11], meaning that it is not transparent how these models reach their decisions. The black box of DNNs is a major issue in life science and drug discovery applications, as further discussed below.

## Data heterogeneity

Life science and drug discovery data are highly heterogeneous in terms of volumes, composition and complexity. Early-phase drug discovery concentrating on target validation, bioassays, compounds and activity data is not a data-rich discipline compared with other areas where DL has made a strong impact. In early-phase drug discovery, data sets from medicinal chemistry are typically confined to test results for individual or parallel compound series and therefore limited in size. This also applies to data sets from, for example, probe investigations in chemical biology, time series experiments in biology or confirmatory assays in biological screening. A consequence of data heterogeneity and sparseness is that sufficiently large data sets for ‘hungry’ DNNs are often not available. Moreover, informatics approaches in the life sciences have traditionally employed pre-defined object (for example, target or compound) representations (descriptors) and not relied on representation learning.

## Relative performance & new opportunities

Given data heterogeneity in sparseness in the life sciences and drug discovery, only relatively small sets of structured training data are often available for model building. These conditions do not play into the strengths of DL, as discussed above. As a consequence, currently available benchmark studies indicate that DNNs often reach the performance of simpler ML methods in standard applications such as molecular property predictions, but do not significantly exceed it. Such observations only provide weak support for using highly complex DNNs for predictions tasks in the life sciences and drug discovery.

Importantly, however, DL has enabled a number of applications that have so far been difficult or impossible to address using ML, which represents a major attraction. Among others, topical examples include chemical reaction modeling [12,13], generative compound design [14] or *de novo* protein structure prediction [15]. In these cases, some unprecedented advances have recently been made. In addition, molecular representation learning is increasingly explored in drug discovery in cases where sufficient data are available [16]. However, whether or not newly learned representations will lead to general performance improvements of DL compared with established pre-defined descriptors remains to be seen. Currently, no firm conclusions can be drawn.

## Model impact & acceptance

Currently, practical applications of DL are still rare in medicinal chemistry and drug discovery [17]. Ultimately, DL will only become an integral part of interdisciplinary research if it measurably impacts experimental programs. This can be achieved, for example, by significantly reducing design–test–make–analyze (DMTA) cycle times or, more importantly, by strongly contributing to the discovery or generation of small molecules or biologicals with new or improved functional properties, more selective chemical probes or novel drug candidates. Importantly, demonstrated advances to further establish DL in interdisciplinary settings are only possible if life science investigators and drug discovery practitioners agree to rely on predictions for experimental design. Compared with the current situation, this will require further increased model acceptance in interdisciplinary settings. As is the case with any new technology, time will be required until DL can realize its full potential in this area. However, there are specific requirements that must be met to further increase the confidence of experimentalists in predictive models.

## Rationalizing predictions

Experimentalists are naturally reluctant to rely on predictions that are difficult or impossible to understand. Given the black box nature of DNNs, this presents a major challenge for the acceptance of complex DL models for experimental design. Therefore, increasing attention is being paid to model-agnostic approaches for ‘explainable AI’ (XAI) that make it possible to rationalize the outcome of predictions and interpret them in chemical or biological terms [18,19]. Among others, these approaches include feature weighting or selection methods to identify representation features making largest contributions to individual predictions or other methods that determine feature subsets minimally required to arrive at a correct (or incorrect) prediction [19]. In medicinal chemistry, such feature subsets might define structural patterns that can be interpreted by mapping them on test compounds.

## Quantifying prediction uncertainty

Closely related to such XAI approaches are methods that quantify the uncertainty of predictions. Obtaining uncertainty estimates also helps to build confidence in predictive modeling and further supports model acceptance for planning experiments. Although there are ML methods that provide inherent prediction uncertainties such as Gaussian process modeling [20], most ML/DL methods including DNNs produce numerical end points without uncertainty estimates. Approaches for uncertainty quantification of predictions complementing ML/DL include probabilistic [21] and ensemble methods [22]. The latter methods determine prediction variance on the basis of differently trained models generated with the same algorithm. For probabilistic approaches, Bayesian DNNs provide a prime example [21]. However, due to their computational costs, Bayesian DNNs are only applicable to large data sets if approximations are introduced.

## Data sparseness

In the presence of data sparseness, as discussed above, DL benefits from complementary learning strategies that are designed to limit the number of required training instances [23]. However, whether or not DL in combination with such learning strategies yields significant performance increases over standard ML methods in practical applications also remains to be determined. Currently available benchmarks do not provide a clear picture.

## Prospective applications

The ultimate assessment of the potential of DL for life science and drug discovery research depends on prospective applications, that is, predictions that are experimentally tested in ‘real’ projects (rather than benchmark-type settings). This requires that experimentalists are willing to accept predictions to guide their work, as discussed above. Moreover, confidence in predictive modeling is essential to rely on predictions in high-priority projects where impact is critically important and highly valued. Currently, a number of AI companies claim successes in proprietary drug discovery projects, but scientifically rigorous reports of such case studies are rare. In prospective applications, it must also be considered that predictions relying on project data also assess data generation processes and data integrity [24] and that predictions might succeed or fail for different reasons. Accordingly, an incorrect prediction might not necessarily be attributable to methodological failure. Hence, in prospective applications, predictions should be analyzed, explained and carefully evaluated within a given project context and care should be taken not to prematurely generalize successes of failures.

## Conclusion

The rise of AI in many scientific fields comes along with promises and new challenges. In most cases, AI refers to DL, which is only a part of methodological spectrum of AI. In the life sciences and drug discovery, deep learning will ultimately only live up to expectations if it impacts experimental research and development efforts. This will require time and no immediate breakthroughs should be expected, especially not in drug discovery, given the long drug development times. For example, medicinal chemists currently witness exciting methodological advances through DL in synthesis design, but frequently point out that no easily accessible DL tools are available at present to support their day-to-day efforts in compound optimization. Thus, transforming expert-dependent complex DNN models into robust and widely usable computational tools will be one of the grand challenges going forward. Moreover, increasing numbers of prospective applications will be essential to further advance DL in interdisciplinary research and demonstrate true impact on high-profile projects. In turn, this will require narrowing the gap between DL and experiments and further increasing the confidence of practitioners to rely on computational predictions. The sooner these challenges are tackled, the better – not only for DL and its proponents, but also for the life science arena as a whole.
